# Books Reviewed

**Published:** 1866

**Authors:** 


### Books Reviewed.

Circular No. 6, War Department, Surgeon General's Office, Washington, D. C., November 1, 1865. Reports on the Extent and Nature of the materials available for the preparation of a Medical and Surgical History of the Rebellion. Printed for the Surgeon General's Office, by J. R. Lippincott & Co. Philadelphia; 1865.

This Report comprises two communications to the Surgeon General. The first, relating to the surgical department, was prepared by George A. Otis, Brevet Lieut. Col. and Surgeon U. S. Vols.; and the second, under the direction of J. J. Woodward, Ass't Surgeon and Brevet Major U. S. A., relating to the medical department.

"The material relating to the surgery consists of reports from the various medical officers, and of illustrations of pathological specimens, drawings and models." The extent of the material available is, in the words of Dr. Otis, "*simply enormous*," and although the time has not yet arrived when we can, with any accuracy, determine the number of wounded, yet from the reports of about one-half of the regiments, their numbers amounted to 187,-470, while the number of wounded of the French and English armies in the Crimean war amounted to 51,962. Again, in comparing the cases of some of the major operations, "as the excision of the head of the humerus," with the Crimean returns, we find 16 of these excisions reported in the English, 38 in the French, and 575 in our own army.

In the earlier part of the war an army medical museum was established, and the medical staff was invited to forward preparations to it. So general was the response, that in January, 1862, a numerical list of 1248 surgical and pathological specimens was published. These contributions steadily increased, so that the museum has now already 5480 surgical specimens in its possession, not only of recent injuries, but of illustrations of reparative processes after injuries and morbid process, so that numerically, at least, this institution is richer than either those of France or England. Furthermore, some artists and a colorist were connected with the museum, to prepare preparations of surgical pathology and representations of remarkable injuries, and of lesions of the viscera in different diseases. Subsequently, a photograph gallery was also established, and the museum is now in possession of 1000 photographic representations of wounded and mutilated men.

The defects of the old monthly blanks of "sick and wounded," was felt very early, and a new form was determined upon, by which the regional classification of wounds was carried out to a far greater perfection, thus enhancing the value of the statistics very much. The recorded gun-shot wounds of the head are 5046, which have been divided into two classes; first, gun-shot fractures of the cranium and contusions of the skull, resulting in lesions of the encephelon; and secondly, simple contusions and flesh wounds of the skull. Under the first class 1104 cases were recorded. In 107 of these the trephine was employed, the result being 60 deaths and 47

recoveries. In 114 cases foreign substances and speculi of bone were removed by the elevator without the use of the trephine; of these 61 died and 53 recovered. The foregoing figures give us a ratio of 43.3 per cent., while in 483 cases treated by expectancy, the ratio of recovery being only 20.5 per cent. Yet the favorable result which has been exhibited in cases in which operative procedure was instituted will be greatly modified when the results of that great proportion of the field operations of trephining shall have been ascertained. Of 22 specimens of gun-shot contusions at the army medical museum, either "necrosis has taken place, with exfoliation of the external table only, or of the entire thickness of the bone, or else inflammatory suppuration occurred in the deploe or between the skull and the dura mater."

The result of 1272 penetrating gun-shot wounds of the chest have been ascertained, of these 930 proved fatal, or 73 per cent. The antiphlogistic treatment in this class of wounds appears to have been almost entirely abandoned. The hæmorrhage was treated by cold applications, and the pain allayed by the administration of opium in some form. In no instance was the operation of paracentisis deemed necessary for the evacuation of effusions of blood, and the practice of "hermetically sealing" wounds of this character, have been found entirely unwarrantable by the results obtained.

The recoveries from penetrating gun-shot wounds of the abdomen was 26 per cent., and although this percentage is unexpectedly large, yet only such cases are reported in which the diagnosis was beyond a doubt. In many instances fæcal fistula were produced which would however after some lapse of time close without operative procedure. Gun-shot injuries of the illeum and of jejunum proved far more fatal than those of the large intestines. In none of the cases reported was it deemed advisable to apply a ligature to the intestine. Of 32 recorded cases of wounds of the liver only four terminated favorably. These wounds were usually followed by large extravacations into the abdominal cavity, giving rise to rapidly fatal peritonitis. But few cases of recovery are reported after protrusion of the abdominal viscera. "In two cases in which loops of small intestine protruded from the wound, they were immediately returned and retained by adhesive straps and bandages, the patients recovering with ventral hernia." The cases of gun-shot wounds of the bladder have all proved fatal, excepting those in which the injury was of that part of the viscus uncovered by peritoneum.

We regret that space does not permit us to lay any more extracts from these statistics before our readers, and before concluding will hastily notice the second part of the report.

The medical department under the direction of Dr. J. J. Woodward, furnishes us with numerous interesting facts upon hospital organizations and upon the nature of diseases of armies. From his report it appears that during the war 202 general hospitals were in operation at one time with an accommodation for 136,804 patients. During the first year of the war the average strength of our army present was 281,177 men, and the average strength constantly present in hospitals was 9,759, making the total strength of the army 290,936 men, among which 14,183 deaths from disease occurred. This number of deaths, large as it is, does not represent the actual rate of mortality, as the reports for the first year were very incomplete. The mean strength present for duty for the second year was 598,821 men, with an average attendance in hospital of 45,687, making the total strength of the army 644,508, the rate of mortality from disease being 42,000 men. The mortality rates for the last half of the war have not been as yet completed, but it is believed that the proportionate rate of deaths will be less than for the first half.

From the foregoing figures the mortality of our armies from disease for the first half of the war appears to be 55.45 per 1000 mean strength, while the mortality of our troops in the Mexican war was 103.5 per 1000, and that of the English and French armies in the Crimea being respectively 232 and 300 per 1000 mean strength. Dr. J. J. Woodward ascribes this favorable result as a consequence of the liberality of the commissary and medical supplies, the careful exclusion of men unfit for military life, and the superior hygienic advantages of the greater part of the general hospitals.

The medical staff which served in the late war was composed "of a Surgeon General, one Assistant Surgeon General, a Medical Inspector General, 16 Medical Inspectors, 170 Surgeons and

Assistant Surgeons of the regular army, 362 volunteer Staff Surgeons and Assistant Surgeons, 3000 regimental Surgeons and Assistant Surgeons of Volunteers, 2500 Acting Assistant Surgeons or Physicians serving under contract, and 6 Medical Store-keepers."

We have laid before our readers a brief synopsis of the treasures contained in the Surgeon General's office, and we shall look forward to the volumes on the surgical and on the medical history of the war, soon to be published, with the greatest interest.

---

On Wakefulness, with an Introductory Lecture on the Philosophy of Sleep. By William A. Hammond, M. D., etc.

This work has previously been received essentially in its present style, but in pamphlet form, and was then noticed. We now offer our thanks to the publishers for favoring us with it, in the present elegant and permanent form. We can favor our readers with one quotation only upon the treatment of wakefulness:

"Among the more purely medicinal agents, *bromide of potassium* occupies the first place, and can almost always be used with advantage to diminish the amount of blood in the brain, and to allay any excitement of the nervous system that may be present in the sthenic form of insomnia. That the first named of these effects follows its use, I have recently ascertained by experiments upon living animals, the details of which will be given at another time. Suffice it now to say, that I have administered it to dogs whose brains had been exposed to view by trephining the skull, and that I have invariably found it to lessen the quantity of blood circulating within the cranium, and to produce a shrinking of the brain from this cause. Moreover, we have only to observe its effects upon the human subject to be convinced that this is one of the most important results of its employment. The flushed face, the throbbing of the carotids and temporals, the suffusion of the eyes, the feeling of fullness in the head, all disappear as if by magic under its use. It may be given in doses of from ten to thirty grains—the latter quantity is seldom required, but may be taken with perfect safety in severe cases."

